# Identification and Molecular Characterization of the Chloroplast Targeting Domain of *Turnip yellow mosaic virus* Replication Proteins

**DOI:** 10.3389/fpls.2017.02138

**Published:** 2017-12-19

**Authors:** Lucille Moriceau, Lucile Jomat, Stéphane Bressanelli, Catherine Alcaide-Loridan, Isabelle Jupin

**Affiliations:** ^1^Laboratory of Molecular Virology, Institut Jacques Monod, CNRS, Université Paris-Diderot, Paris, France; ^2^Université Paris-Sud – Université Paris-Saclay, Orsay, France; ^3^Institute for Integrative Biology of the Cell, CEA, CNRS, Université Paris-Sud – Université Paris-Saclay, Gif-sur-Yvette, France

**Keywords:** RNA viruses, TYMV, viral replication, replication protein, viral replication complexes, membrane targeting, chloroplast envelope membrane, amphipathic helix

## Abstract

*Turnip yellow mosaic virus* (TYMV) is a positive-strand RNA virus infecting plants. The TYMV 140K replication protein is a key organizer of viral replication complex (VRC) assembly, being responsible for recruitment of the viral polymerase and for targeting the VRCs to the chloroplast envelope where viral replication takes place. However, the structural requirements determining the subcellular localization and membrane association of this essential viral protein have not yet been defined. In this study, we investigated determinants for the *in vivo* chloroplast targeting of the TYMV 140K replication protein. Subcellular localization studies of deletion mutants identified a 41-residue internal sequence as the chloroplast targeting domain (CTD) of TYMV 140K; this sequence is sufficient to target GFP to the chloroplast envelope. The CTD appears to be located in the C-terminal extension of the methyltransferase domain—a region shared by 140K and its mature cleavage product 98K, which behaves as an integral membrane protein during infection. We predicted the CTD to fold into two amphipathic α-helices—a folding that was confirmed *in vitro* by circular dichroism spectroscopy analyses of a synthetic peptide. The importance for subcellular localization of the integrity of these amphipathic helices, and the function of 140K/98K, was demonstrated by performing amino acid substitutions that affected chloroplast targeting, membrane association and viral replication. These results establish a short internal α-helical peptide as an unusual signal for targeting proteins to the chloroplast envelope membrane, and provide new insights into membrane targeting of viral replication proteins—a universal feature of positive-strand RNA viruses.

## Introduction

Positive-strand RNA [(+)RNA] viruses—the largest class of viruses, include significant pathogens of humans, animals, and plants (King et al., 2012). Replication of their genome requires the assembly of an intricate viral replication complex (VRC) comprising both viral and host proteins (reviewed in Nagy and Pogany, 2011; Wang, 2015).

A universal feature of (+)RNA VRCs is their close association with intracellular membranes (Buck, 1996; Salonen et al., 2005; Grangeon et al., 2012), resulting in massive viral-induced membrane rearrangements and/or proliferation. These host-derived membranes, which anchor the components of the replication complex, are thought to create a favorable environment for RNA synthesis by concentrating crucial viral and host factors, and possibly protecting the viral RNA progeny from host cell antiviral surveillance system.

Strikingly, there is a great diversity in the origin of membranes or organelles selected for the assembly of VRCs, as different families of (+)RNA viruses have the ability to capture either the endoplasmic reticulum (ER), Golgi apparatus, vacuole, mitochondria, peroxisomes, lysosomes, or chloroplasts (reviewed in Netherton et al., 2007; Laliberté and Sanfaçon, 2010; Verchot, 2011).

Despite great advances in imaging of these subcellular structures, characterisation of their ultrastructural details, and identification of some of the host factors or cellular pathways involved in their formation, all of which has revealed many similarities among (+)RNA VRCs (reviewed in den Boon and Ahlquist, 2010; Belov and van Kuppeveld, 2012; de Castro et al., 2013; Harak and Lohmann, 2015), we are still far from understanding the molecular details of this membrane association, and how viral replication factors target, bind, and remodel membranes of specific cell organelles during VRC biogenesis.

As discussed in den Boon et al. (2010), among these unresolved questions are « what are the detailed molecular mechanisms by which specific viruses target their replication factors and their RNAs to particular membranes or other intracellular sites to assemble replication complexes or factories ? », and « how do different viruses orchestrate the varied and often complex membrane rearrangements associated with their replication processes ? ».

**FIGURE 1:**
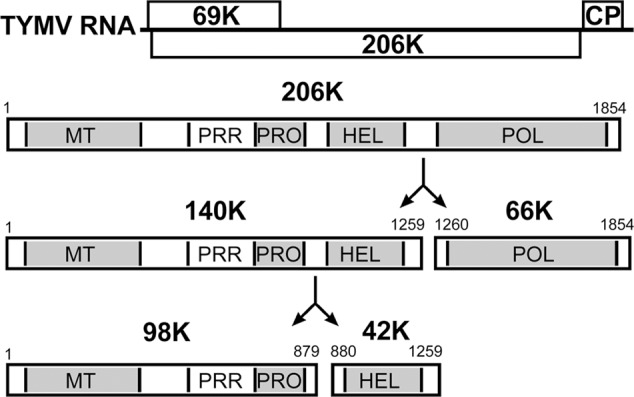
Schematic representation of the genomic organization of TYMV RNA and 206K protein processing. The open reading frames are indicated by open rectangles, and correspond to the 69K suppressor of RNA silencing and movement protein, the 206K replication polyprotein and the coat protein (CP). Protein functional domains of the encoded 206K protein are indicated. 206K is proteolytically processed at peptide bonds 1259–1260 (HEL↓POL) to generate 140K and 66K. The former is further processed at peptide bonds 879–880 (PRO↓HEL) to generate 98K and 42K (Jakubiec et al., 2007).

Here, we address the question of VRC targeting using *Turnip yellow mosaic virus* (TYMV), a (+)RNA plant virus that shares replication features with other viruses in the alphavirus-like supergroup (Goldbach and Wellink, 1988; Koonin and Dolja, 1993) and has proven useful in the study of fundamental aspects of viral multiplication (Dreher, 2004). The 6.3-kb genomic RNA of TYMV encodes two extensively overlapping open reading frames (ORFs) (**Figure 1**), producing a 69K protein that serves as the viral movement protein and viral suppressor of RNA silencing, and a 206-kDa precursor protein (206K) that is the only viral protein necessary for replication (Weiland and Dreher, 1989). The 206K protein contains sequence domains indicative of methyltransferase (MT), proteinase/deubiquitinase (PRO), NTPase/helicase (HEL), and RNA-dependent RNA polymerase (POL) activities, as well as a proline-rich region (PRR) between the MT and PRO domains (**Figure 1**). Previous studies have demonstrated the involvement of the PRO domain in the cleavage of 206K, giving rise to an N-terminal product of 140 kDa (140K) and a C-terminal 66-kDa protein (66K) encompassing the POL domain (Bransom et al., 1996; Prod’homme et al., 2001). 140K can then be further cleaved to release 98K, which contains the MT, PRR, and PRO domains, and a 42-kDa protein (42K) encompassing the HEL domain (Jakubiec et al., 2007) (**Figure 1**).

TYMV replication occurs in close association with the chloroplast outer envelope membranes, which are subject to extensive alterations upon infection, including the formation of membrane invaginations—or spherules—that host the VRCs (Ushiyama and Matthews, 1970; Hatta et al., 1973; Prod’homme et al., 2001). The 140K protein was previously shown to play a key role in the assembly of TYMV replication complexes, as it is responsible for targeting the TYMV replication complexes to the chloroplast envelope membrane (Prod’homme et al., 2003) and allows the recruitment of 66K polymerase to the replication sites through defined protein–protein interactions between the PRO and POL domains (Jakubiec et al., 2004). Whether cleavage of the precursor 140K into mature 98K and 42K proteins occurs before or after chloroplast membrane targeting is presently unknown; thus, for the sake of simplicity, herein we refer to the 140K/98K protein as being the protein entity that is targeted to the chloroplasts. So far, the determinants for subcellular localization and membrane interaction of the 140K/98K protein have not been defined.

In this study, we investigated the mode of membrane association of VRCs during infection, as well as the determinants for the subcellular localization of the 140K/98K protein to the chloroplast envelope using transient expression of EGFP fusion proteins in plant cells and observation by confocal microscopy.

Deletion studies identified a minimal internal domain of 41 amino acid residues, which is sufficient for chloroplast targeting. This region was predicted to fold into amphipathic helices, which was confirmed by circular dichroism analysis of a synthetic peptide. Disruption of the helical structure, or alterations of the hydrophobic face, were shown to affect chloroplast targeting and membrane association *in vivo*, and to have deleterious effects on viral RNA replication, indicating that the integrity of these amphipathic helices is essential for an early function in the viral life cycle, and demonstrating their key role in the targeting of TYMV replication complexes to chloroplast envelope membranes.

## Materials and Methods

### Plasmid Constructs

All DNA manipulations were performed using standard cloning techniques (Sambrook et al., 1989), or using the Gibson assembly method (Gibson et al., 2009).

Plant expression vectors were derived from pΩ-EGFP-140K (Prod’homme et al., 2003) or pΩ-98K [formerly designated as pΩ-140K(1-879)] (Jakubiec et al., 2007). Mutations were introduced by PCR-mediated site-directed mutagenesis or by subcloning of restriction fragments. The overall structures of all plasmids were confirmed by restriction analysis, and the sequences of PCR-generated DNA fragments were confirmed by DNA sequencing. When proteins are truncated, the encoded amino acids are indicated within parentheses in the plasmid name. Primer sequences and cloning details will be made available on request.

To generate a chloroplast-specific subcellular marker, the eqFP670 fluorochrome (hereafter named NiRFP)—a bright and highly photostable fluorescent protein that fluoresces in the near infra-red (ex 605 nm; em 670 nm) (Shcherbo et al., 2010)—was fused in frame with the N-terminal signal peptide of the small subunit of ribulose-1,5-diphosphate carboxylase (RbcS) as a synthetic construct obtained from Shanghai ShineGene Molecular Biotech, Inc. (Shanghai, China). The corresponding gene fusion was then cloned into the transient expression vector pΩ (Prod’homme et al., 2003) to generate pΩ-RbCS-NiRFP.

The full-length TYMV cDNA clone E17, which produces infectious transcripts, and its derivative E17-stop69K, in which the 69K is truncated at amino acid 30 without modification of the 206K ORF were described previously (Drugeon and Jupin, 2002; Prod’homme et al., 2003). Point mutations in the αA and αB helices were introduced into E17-stop69K by subcloning from the pΩ-EGFP-140K mutant constructs. Mutant E17-G404R, carrying a mutation within the ultra-conserved GDD motif in the polymerase catalytic domain (Jakubiec et al., 2006), served as a negative control.

To generate bimolecular fluorescence complementation (BiFC) expression vectors, the N- (nYFP; amino acids 1–174) and C- (cYFP; amino acids 175–239) termini of the yellow fluorescent protein (YFP) were PCR-amplified and subcloned into the expression vectors pΩ-EGFP-66K (Prod’homme et al., 2003) and pΩ-EGFP-98K (Jakubiec et al., 2007), to generate pΩ-nYFP-98K and pΩ-cYFP-66K, respectively. Point mutations were then introduced into pΩ-nYFP-98K by subcloning. Expression vectors pΩ-nYFP-REL and pΩ-cYFP-REL encoding Renilla luciferase fused to nYFP or cYFP, respectively, were obtained from Shanghai ShineGene Molecular Biotech, Inc. and used as negative controls, whereas pΩ-YFP, in which full-length YFP was cloned into the transient expression vector pΩ (Prod’homme et al., 2003) was used as a positive control.

### Preparation and Transfection of Arabidopsis Protoplasts

Protoplasts of *Arabidopsis thaliana* were prepared and transfected with 1–15 μg of plasmids or *in vitro* transcripts as described previously (Camborde et al., 2010), with minor modifications (Planchais et al., 2016). pΩ-RbCS-NiRFP was used as a chloroplast subcellular marker and was co-transfected with constructs encoding proteins fused to EGFP. Where applicable, samples were supplemented with the control vector pΩ-REL encoding Renilla luciferase (Camborde et al., 2010) to keep the total amount of nucleic acids transfected constant. Capped *in vitro* transcripts were generated from linearized DNA templates as described previously (Drugeon and Jupin, 2002).

### Analysis of the Association of TYMV Replication Proteins with Membranes

Chinese cabbage (*Brassica pekinensis* cv. Granaat) plants were grown and inoculated with TYMV as described previously (Prod’homme et al., 2001). At 4–6 weeks post-inoculation, plants were kept in the dark for 1 day before being harvested in order to minimize the accumulation of starch. The young developing leaves from the center of the rosette (1 g of fresh weight) were collected and ground in a mortar and pestle with 2.5 ml of extraction buffer (Camborde et al., 2007), followed by filtration through four layers of cheesecloth. Membrane fractions were collected by centrifugation at 25,000 × *g* for 30 min at 4°C over a cushion of buffer G (25 mM Tris-HCl pH 7.5, 7.5 mM MgCl_2_, 0.5 mM EDTA, 20% glycerol, 2 mM DTT), and resuspended in 1.2 ml of 1.25x buffer G containing a mixture of protease inhibitors (Complete protease inhibitor cocktail, Roche); 400 μl of resuspended membranes were then mixed with 100 μl of 5 M NaCl, 5 M KCl, 2.5% Lubrol W1, 0.5 M Na_2_CO_3_ or 0.5 M NaOH and were incubated for 1 h at 4°C with occasional gentle agitation. Samples were then centrifuged at 100,000 × *g* for 30 min at 4°C to collect supernatant and pellet fractions, which were resuspended in buffer G. For urea treatments, membrane fractions were resuspended in 0.75 ml of 2x buffer G containing protease inhibitors; 120 or 240 mg of crystalline ultra-pure urea (Pierce Sequanal grade) were added to 250 μl of resuspended membranes, and the final volume was adjusted to 500 μl with H_2_O to reach a final concentration of 4 or 8 M urea, respectively. After incubation for 1 h at 4°C (4 M urea), or 2 h at RT (8 M urea) respectively, samples were centrifuged at 100,000 × *g* for 30 min at 4°C or 20°C respectively, to collect supernatant and pellet fractions, which were resuspended in buffer G. After addition of Laemmli sample buffer, samples of each fraction corresponding to the same amount of fresh tissue were subjected to SDS-PAGE. Transfected protoplasts were harvested for subcellular fractionation at 24–30 h post-transfection (hpt) as previously described (Prod’homme et al., 2001) with minor modifications. Following a washing step in PBS containing protease inhibitors, 5 × 10^6^ protoplasts were resuspended in 0.5 ml of buffer H (100 mM Tris-HCl pH 7.5, 10 mM KCl, 5 mM MgCl_2_, 1 mM EDTA, 10% glycerol, 0.1% β-mercaptoethanol) containing protease inhibitors, and were lysed by 30 passages through a 23-gauge syringe needle. Cell debris were removed by two successive centrifugations at 500 × *g* for 10 min at 4°C, and the supernatant fraction was further centrifuged at 25,000 × g for 1 h at 4°C to collect pellet and supernatant fractions (P25 and S25, respectively). The P25 pellet was subjected to a washing step by resuspension in H buffer, and additional centrifugation at 25,000 × *g*. Proteins in the P25 pellet were resuspended in buffer H. After addition of Laemmli sample buffer, samples of each fraction corresponding to the same amount of fresh tissue were subjected to SDS-PAGE.

### Antibodies, Immunoprecipitation, and Immunoblotting Experiments

Total protein extraction from protoplasts, SDS-PAGE, immunoblotting and detection of viral proteins were performed as described (Prod’homme et al., 2001, 2003; Jakubiec et al., 2004, 2007) using nitroblue tetrazolium (NBT)/5-bromo-4- chloro-3-indolylphosphate (BCIP) as a substrate. Polyclonal antisera raised against the TYMV 66K protein, the PRR domain shared by 140K and 98K proteins (hereafter, anti-98K antiserum) and the TYMV capsid were described previously (Prod’homme et al., 2001; Jakubiec et al., 2004), and were used at dilutions of 1/2,000, 1/8,000, and 1/50,000, respectively. Anti-EGFP polyclonal antibody (Abcam Ab290) was used at 1/2,000 dilution. In some instances, the nitrocellulose membranes were probed successively with the anti-66K and anti-98K antisera, and NBT/BCIP and Fast red/Naphtol (Sigma) were sequentially used as substrates to allow dual-color detection of the viral proteins as previously described (Jakubiec et al., 2004).

### Spinning Disk Confocal Laser Microscopy (SPCLM)

Transfected Arabidopsis protoplasts were harvested 48 hpt and were directly observed by transferring 40 μl of cell suspension in one channel of a μ-slide VI channel slide (Ibidi). Confocal images were acquired using a CSU22 spinning head (Yokagawa) mounted on a DMI6000 microscope (Leica) equipped with a Leica 100x/1.4 NA objective. Images with EGFP fluorescence were acquired by using a 491-nm laser line and were collected between 500 and 560 nm. NiRFP fluorescence and chlorophyll autofluorescence were excited with a 635 nm laser line and collected between 600 and 700 nm. Images were captured with a QuantEM 512SC camera (Photometrics) driven by the software Metamorph (Universal Imaging Corp.). They were acquired in sequential mode and digitally superimposed. Color levels were processed and figures assembled using Photoshop CS (Adobe).

### BiFC Experiments

Protein interactions were detected in transfected Arabidopsis protoplasts by detecting complemented YFP using a flow cytometer as previously reported (Berendzen et al., 2012). Transfected protoplasts were harvested at 40 hpt, sedimented at RT for 30 min, and 25 μl of cells were diluted in 250 μl of PBS immediately before being analyzed in a CyAn ADP 9C flow cytometry analyzer (Beckman-Coulter). YFP was excited using a 488 nm argon laser, and fluorescence was detected in channels FL1 (528/38) and FL2 (579/34). After exclusion of cell debris, 10,000–15,000 events were analyzed and the percentage of BiFC-positive cells was obtained by plotting the primary fluorescence channel against the secondary fluorescence channel and selecting cells that had significant shifts in the YFP channel over the autofluorescence. Protoplasts transfected with H_2_O or pΩ-YFP were used to gate the signal in each experiment. To normalize experiments, the percentage of fluorescent cells was corrected from the percentage of transfection, as determined upon transfection of 5 × 10^5^ protoplasts with 5 μg of the pΩ-YFP plasmid.

### Peptide Synthesis

The peptide RSPIASLSLYLRQHWRRLTATAVPILSFLTLLQRFLPLR corresponding to residues 374–409 of the 140K/98K protein—flanked by two Arg residues to improve peptide solubility in aqueous solvent—was synthesized by the company Proteogenix (>98% purity level). Peptide aliquots of 1 mg were resuspended either in 1 ml of 2,2,2-trifluoroethanol (TFE), or in 1 ml of aqueous buffer (10 mM sodium phosphate buffer pH 7.5, 10% glycerol). In the latter case, insoluble material remaining after vortexing was removed by centrifugation at 200,000 × g for 1 h at 4°C, and the concentration of soluble peptide was determined using a Nanodrop spectrophotometer, based on a molar extinction coefficient 𝜀_(280__nm)_ value of 6970 cm^-1^M^-1^, calculated according to Gill and von Hippel (1989). Peptide solutions were stored at -20°C.

### CD Spectroscopy

CD spectra were recorded at 20°C using a Jasco J-810 spectropolarimeter equipped with a 0.1-mm quartz cell (Hellman #106-QS.0.1). Each spectrum was the average of 10 acquisitions recorded in the 280–185 nm range in 1-nm steps, a bandwidth of 1 nm, and a speed of 50 nm/min. The samples were in a total volume of 20 μl in aqueous buffer (10 mM sodium phosphate buffer pH 7.5, 10% glycerol), or 2,2,2-trifluoroethanol (TFE) 100, or 50% TFE-50% aqueous buffer. The peptide concentrations were in the range of 100–200 μM. Corresponding blanks were realized for each assay.

The CD spectral analysis and the predicted percentage of α-helices, β-strands, turns or unordered residues were calculated using the algorithms SELCON3, CONTINLL (van Stokkum et al., 1990) and CDSSTR (Compton and Johnson, 1986), which are available on the Dichroweb server (Whitmore and Wallace, 2008)^1^, using reference set 7 (Janes, 2009).

### Protein Sequence Analyses and Structure Predictors

To predict chloroplast transit peptides, the TargetP server^2^ (Emanuelsson et al., 2007) was used in the plant mode without cut-offs, including cleavage site prediction.

Protein sequence analyses and secondary structure predictions were performed using the algorithms DPM (Deléage and Roux, 1987), DSC (King and Sternberg, 1996), GOR4 (Garnier et al., 1996), HNNC (Guermeur et al., 1999), Predator (Frishman and Argos, 1996), SIMPA96 (Levin, 1997) and SOPM (Geourjon and Deléage, 1994), which are available on the integrated server NPS@ (Combet et al., 2000) ^3^, PSI-PRED (McGuffin et al., 2000)^4^, or PEP-FOLD (Camproux et al., 2004)^5^.

Transmembrane helix predictions were performed using TMHMM (Krogh et al., 2001)^6^, TM-Pred ^7^, DAS-TM (Cserzo et al., 2004)^8^, HMMTOP (Tusnády and Simon, 2001)^9^, and SOSUI (Hirokawa et al., 1998) ^10^.

Helical wheel predictions were performed using Heliquest (Gautier et al., 2008)^11^.

*De novo* peptide modeling was performed using PEP-FOLD3 (Lamiable et al., 2016)^5^, and structures were represented by PyMOL, using the same color code as Heliquest.

Predictions of lipid modifications and glycosylphosphatidylinositol (GPI) anchor were performed using PredGPI (Pierleoni et al., 2008)^12^ and GPS lipid (Xie et al., 2016)^13^.

### RNA Isolation and cDNA Synthesis

Arabidopsis protoplasts were transfected with each viral mutant between 6 and 10 times in two independent experiments using various batches of *in vitro* transcripts. Transfected protoplasts were collected 48 hpt by centrifugation at 80 × *g*, immediately frozen in liquid nitrogen and stored at -80°C. Total RNA extraction and cDNA synthesis were performed as previously described (Jakubiec et al., 2006; Jupin et al., 2017)

### Real-time qPCR Amplification and Quantification of Viral RNA Accumulation

Real-time quantitative PCR (qPCR) reactions were performed in 384-well plates with a LightCycler 480 Real-Time PCR system (Roche) as described (Jupin et al., 2017). Relative quantities of cDNAs were calculated and normalized as described (Vandesompele et al., 2002; Hellemans et al., 2007), using *EF1α (*At5g60390) and *PDF2 (*At1g13320) as reference genes (Lilly et al., 2011). Data relative to mutant transcripts were then expressed as a percentage of the mean value of the data obtained with control E17-stop69K transcripts that were transfected simultaneously and analyzed by qPCR in the same run (Supplementary Table 1).

## Results

### Membrane Association of TYMV Replication Proteins during Viral Infection

**FIGURE 2:**
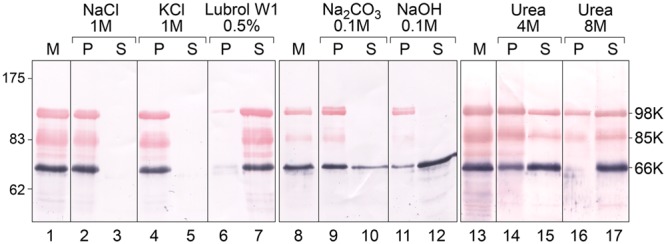
Analysis of the association of TYMV replication proteins with membranes by ionic, alkaline and urea extraction. Membrane fractions (M) were obtained from TYMV-infected Chinese cabbage tissues and were incubated in medium containing 1 M NaCl (lanes 2–3); 1 M KCl (lanes 4–5); 0.5% Lubrol W1 (lanes 6–7); 0.1 M Na_2_CO_3_, pH 11 (lanes 9–10), 0.1 M NaOH (lanes 11–12), 4 M urea (lanes 14–15) or 8 M urea (lines 16–17). Soluble (S) and insoluble pellet (P) fractions were then separated by centrifugation, and each fraction was subjected to 8% SDS-PAGE and immunoblot analysis. Protein samples were revealed sequentially using anti-66K and anti-98K antisera and NBT/BCIP (purple) and Fast Red/Naphtol (red) substrates, respectively. Lanes 1–7, lanes 8–12, and lanes 13–17 correspond to different tissue samples processed and analyzed independently. Molecular mass markers (Biolabs) are indicated on the left, whereas positions of the viral proteins 98K, 85K, and 66K are indicated on the right.

To test the membrane association properties of TYMV replication proteins during viral infection, TYMV-infected Chinese Cabbage tissues were fractionated by centrifugation to recover a membrane pellet fraction containing VRCs (Prod’homme et al., 2001; Jakubiec et al., 2004), which was then analyzed by Western blot using specific antibodies raised against the 66K protein (Prod’homme et al., 2001) and the PRR domain shared by 140K and 98K proteins (Jakubiec et al., 2004) (**Figure 1**). As shown in **Figure 2** (lanes 1, 8, and 13) and consistent with our previous reports, both 66K and 98K—corresponding to the mature N-terminal cleavage product of 140K—were detected in the membrane pellet from infected tissues (Jakubiec et al., 2004, 2007), as well as 85K—a cleavage product previously shown to result from non-specific proteolytic degradation of 98K (Jakubiec et al., 2007).

The mode of membrane association of each replication protein was then investigated by treating the membrane pellet fraction with different compounds, in order to discriminate between integral membrane proteins that are embedded in the phospholipid bilayer, and peripheral membrane proteins, which are attached to membranes by electrostatic interactions with membrane-integral proteins or phospholipid head groups (Singer and Nicolson, 1972; Steck, 1974).

**Figure 2** shows the pellet (P) and soluble (S) fractions of the membrane (M) fraction following extraction with the compounds indicated. Both the 98K and 66K proteins remained attached to membranes upon treatment with 1 M NaCl or 1 M KCl (lanes 2–5), conditions that extract peripheral membrane proteins due to the increased ionic strength of the buffer (Steck and Yu, 1973). Moreover, the 98K protein was still detected in the pellet fraction when membranes were extracted using strong alkaline treatments (0.1 M NaOH or 0.1 M Na_2_CO_3_, pH 11.5) (**Figure 2**, lanes 9–12). As alkaline treatments have been reported to convert closed vesicles into open membrane sheets, and to release soluble proteins that are trapped inside membranous vesicles (Fujiki et al., 1982), the resistance of 98K to alkaline extraction therefore argues against a peripheral association of 98K inside the chloroplast membrane spherules hosting the replication complexes.

The 98K protein was found in the supernatant fraction upon detergent solubilization of the membranes using 0.5% Lubrol W1, a non-ionic detergent used to solubilize TYMV replication complexes (lanes 6 and 7) (Deiman et al., 1997; Jakubiec et al., 2004; Camborde et al., 2007), suggesting that hydrophobic, rather than electrostatic, interactions are the primary 98K membrane association determinants. After extraction of the membrane fraction with 4 M or 8 M urea, treatments which are unable to release transmembrane proteins (Grunfeld et al., 1985; Peiró et al., 2014), a substantial proportion of 98K was found in the supernatant fractions (lanes 14–17), indicating that although 98K associates tightly with membranes, it most likely does not span membranes. Neither acylation sites nor phosphoinositide anchoring are predicted in the TYMV 98K protein sequence (Pierleoni et al., 2008; Xie et al., 2016), making membrane association of the protein through a lipid anchor unlikely.

In contrast, 66K was observed as being partly solubilized by alkaline treatments (lanes 9–12). As 66K was reported to be a soluble protein recruited to the replication complexes via a protein–protein interaction with the PRO domain of 140K/98K (Jakubiec et al., 2004), its localization within the membrane spherules may explain its extractability by alkaline treatments but not high salt treatments. This is consistent with previous immunocytochemistry experiments that reported its localization at the necks of the chloroplast membrane spherules (Prod’homme et al., 2001).

Taken together, these findings provide evidence that the 98K protein behaves as an integral protein embedded in the phospholipid bilayer rather than being peripherally associated with membranes, whereas 66K is most likely a peripheral protein localized within the membrane spherules hosting the replication complexes.

### Subcellular Localization of 140K/98K Protein Deletion Mutants Identifies an Internal 41 Amino Acid Region As the Chloroplast Targeting Domain

We next sought to identify the molecular and structural determinants involved in targeting of the 140K/98K viral replication protein to the chloroplast using deletion mapping. As it is presently unknown whether cleavage of the precursor 140K into mature proteins occurs before or after chloroplast membrane targeting, such determinants were initially sought within the 140K protein precursor.

It should be noted that no chloroplast targeting transit peptide was identified at the N-terminus of 140K/98K (Emanuelsson et al., 2007), consistent with its localization at the chloroplast outer envelope membrane (Jarvis and Robinson, 2004).

**FIGURE 3:**
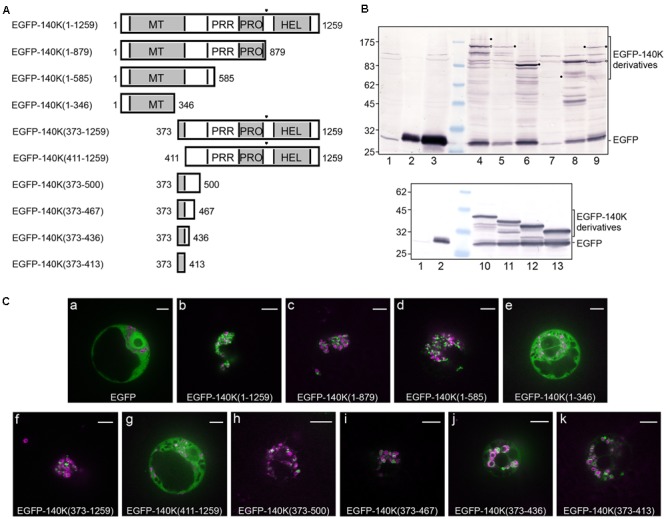
Deletion mapping of the chloroplast targeting domain. **(A)** Schematic representation of the EGFP-140K derivatives. Protein domains are designated as in **Figure 1** and the (PRO↓HEL) cleavage site is represented by a filled triangle. The EGFP moiety present at the N-terminus is not represented. **(B)** Arabidopsis protoplasts were transfected with water (lane 1) or the expression plasmids pΩ-EGFP (lanes 2 and 3), pΩ-EGFP-140K(1–1259) (lane 4), pΩ-EGFP-140K(1–879) (lane 5), pΩ-EGFP-140K(1–585) (lane 6), pΩ-EGFP-140K(1–346) (lane 7), pΩ-EGFP-140K(373–1259) (lane 8), pΩ-EGFP-140K(411–1259) (lane 9), pΩ-EGFP-140K(373–500) (lane 10), pΩ-EGFP-140K(373–467) (lane 11), pΩ-EGFP-140K(373–436) (lane 12) and pΩ-EGFP-140K(373–413) (lane 13). The cells were harvested 48 h post-transfection (hpt) and equivalent amount of total proteins (except lane 2 which corresponds to 1/10th of the other samples) were subjected to 10% SDS-PAGE and immunoblot analysis with anti-GFP antibodies. Molecular mass markers (Biolabs) are indicated on the left, whereas positions of EGFP-140K derivatives and EGFP are indicated on the right. Filled dots indicate the position of full-length proteins, whereas open dots indicate the position of the mature product after processing at the (PRO↓HEL) cleavage site, when appropriate. **(C)** Arabidopsis protoplasts were transfected with the expression plasmids as indicated, together with pΩ-RbCS-NiRFP. Single protoplasts were observed by spinning-disk confocal laser microscopy (SPCLM) 48 hpt and EGFP localization was observed (green). To visualize the localization of chloroplasts, NiRFP fluorescence and chlorophyll autofluorescence were acquired (magenta) and superimposed onto the EGFP fluorescence. Scale bars, 10 μm.

The importance of specific domains of the 140K protein for its subcellular localization was investigated in living plant cells expressing various EGFP-140K deletion mutants (**Figure 3A**), whose expression was verified by western-blotting using anti-EGFP antibody (**Figure 3B**). Targeting of EGFP-140K derivatives to the chloroplast was analyzed by observation of the transfected cells by spinning disk confocal laser microscopy (SDCLM) using EGFP fluorescence to record localization of the viral proteins (green) and chlorophyll autofluorescence/NiRFP fluorescence to record chloroplast localization (magenta) (**Figure 3C**).

Whereas an unfused EGFP moiety was present throughout the cell, staining both the cytoplasm and the nucleus (**Figure 3Ca**), the wild-type EGFP-140K [EGFP-140K(1-1259)] protein was observed localized mainly at the periphery of chloroplasts (**Figure 3Cb**), as previously reported (Prod’homme et al., 2003). Such localization has been shown to be identical to that of the untagged 140K protein as detected by immunofluorescence (Prod’homme et al., 2003). Expression of EGFP-140K also promotes clumping of the chloroplasts, one of the typical cellular perturbations induced by TYMV infection, as previously reported (Prod’homme et al., 2003).

Localization of the EGFP-140K(1-879) (i.e., EGFP-98K) was essentially the same as that of the EGFP-140K protein (**Figure 3Cc**), demonstrating that the chloroplast targeting domain is actually located within the mature 98K protein. The EGFP-140K(1-585) was also targeted to the chloroplast, whereas the EGFP-140K(1-346) protein showed an altered localization, displaying a cytosolic localization (**Figures 3Cd,e**). N-terminal deletion constructs revealed that the EGFP-140K(373–1259) protein was also associated with the chloroplasts, whereas further deletion to residue 411 led to a loss of chloroplast targeting (**Figures 3Cf,g**). From these experiments, we conclude that the region targeting TYMV replication proteins to the chloroplast lies between residues 373 and 585 of 140K/98K proteins.

To further delineate the region involved in chloroplast targeting, additional deletion mutants were expressed in living cells, which all displayed a clear localization around the chloroplasts (**Figures 3Ch–k**) although some staining of the cytosol was more apparent than for full-length EGFP-140K, most likely due to partial release of the EGFP moiety from those fusion proteins (**Figure 3B**, lanes 10–13).

Altogether, these results indicate that the chloroplast targeting region of TYMV replication proteins resides between residues 373 and 413 of the 140K protein, an internal region shared by the 140K and 98K proteins (**Figure 1**). We will therefore refer to this 41-amino acid residues as the 140K/98K “chloroplast targeting domain” (CTD).

### The CTD Contains Predicted Amphipathic α-Helices

**FIGURE 4:**
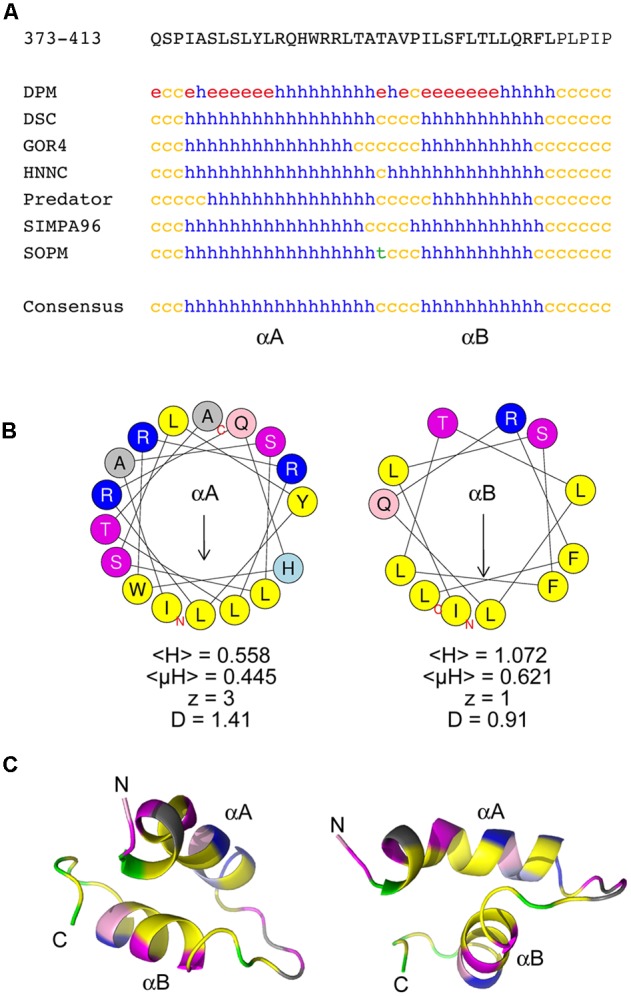
The chloroplast targeting domain contains two predicted amphipathic alpha-helices. **(A)** Sequence and secondary structure predictions of the CTD domain within the 140K/98K protein. The sequence in one-letter code is shown at the top. Secondary structure predictions were made using several predictors from the NPS@ server (Combet et al., 2000). h: helix; c: coil; e: β-sheet; t: turn). The consensus prediction shown below identifies two α-helices designated αA and αB. **(B)** Helical wheel representation of αA and αB helices generated using the HeliQuest server (Gautier et al., 2008), illustrating the strong amphipathic character of both αA and αB. Yellow: hydrophobic residues; purple: serine and threonine residues; dark blue: basic residues; light blue: histidine residues; pink: glutamine residues; gray: other residues. The position of the first (N) and last (C) amino acids of the corresponding peptide sequences are indicated. For each helix, the mean hydrophobicity <H>, the mean hydrophobic moment <μH> (in arbitrary units), the charge z and the discriminant factor D are also indicated. The length of the arrow is proportional to the mean hydrophobic moment <μH>. **(C)** *Ab initio* modeling of the CTD using PEP-FOLD3 (Lamiable et al., 2016). The top model output is displayed as ribbons and colored with the same color code as in **(B)**, with proline residues in green. The position of the first (N) and last (C) amino acids of the corresponding peptide sequences are indicated. Two different views are shown to illustrate the importance of the linker sequence in the orientation of the helices relative to each other.

To gain insight into the structural features of the CTD, the 98K protein was subjected to several protein annotation and secondary structure predictors from the NPS@ server (Combet et al., 2000). As shown in **Figure 4A**, the consensus predicted structure of the CTD was identified as being two α-helices with a short coil linker in between. Similar results were obtained using different protein or peptide secondary structure predictors such as PSI-PRED or PEP-FOLD (McGuffin et al., 2000; Camproux et al., 2004), although the boundaries of the predicted α-helices may differ by a few residues (Supplementary Figure 1). Hereafter, these two α-helices are referred to as αA and αB, respectively.

Whether these helices could correspond to putative membrane-spanning regions was explored using various predictors. Although regions including αB, or part of it, were identified by some predictors as possibly corresponding to a membrane-spanning helix, such predictions appeared to lack consistency (Supplementary Figure 1).

We next explored whether αA and αB could constitute amphipathic helices. Indeed, wheel projection of αA and αB using HeliQuest predictor (Gautier et al., 2008) (**Figure 4B**) revealed an asymmetric distribution of hydrophobic and hydrophilic amino acids on opposite sides of these putative helices—a property known as amphipathy, as confirmed by the calculation of the hydrophobic moment <μH> (Eisenberg et al., 1982). Interestingly, the hydrophobic side of each helix comprises as many as four Leu or Ile residues, which are particularly important residues for membrane anchorage (Granseth et al., 2005). Accordingly, based on the calculation of their discriminant factor D (Gautier et al., 2008), these helices were indeed predicted to have lipid-binding (αA, D > 1.34) or possible lipid-binding (αB, 0.68 < D < 1.34) abilities, respectively.

Such structure predictions were further supported by performing *ab initio* peptide structure modeling using PEP-FOLD3 (Shen et al., 2014; Lamiable et al., 2016). The top five models appeared highly convergent (Supplementary Figure 1), and were consistent with the folding of the CTD into two α-helices bearing hydrophobic faces (**Figure 4C**). Such modeling also revealed that the linker sequence connecting the two helices may play an important role in their positioning relative to each other, as the presence of a proline kink (in green) within the linker may induce local structural constraints, possibly affecting bending of the peptide.

**FIGURE 5:**
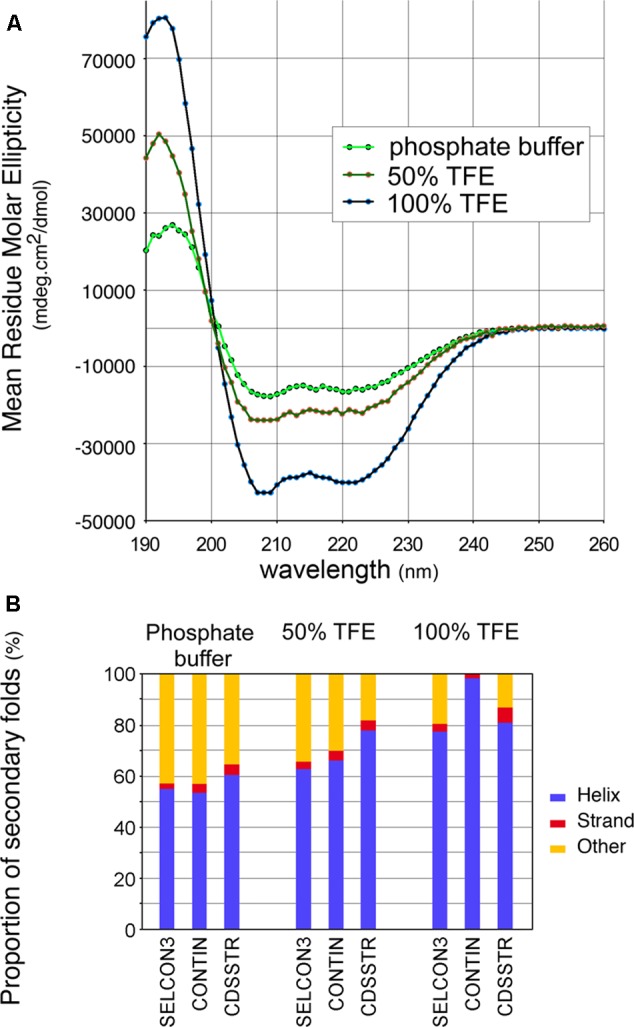
Circular dichroism (CD) spectra of a synthetic peptide confirms the helical conformation of the CTD. **(A)** CD spectra of a synthetic peptide corresponding to residues 374–410 were recorded in aqueous phosphate buffer, in semihydrophobic (50% TFE), and hydrophobic (100% TFE) environments. Each spectrum corresponds to the average of 10 acquisitions. **(B)** Estimated distribution of secondary folds in the synthetic peptide in the different environments assayed, using the deconvolution algorithms SELCON3, CONTIN and CDSSTR from the Dichroweb server (Whitmore and Wallace, 2008).

To confirm the predicted high helical content of the CTD, an ultrapure peptide corresponding to residues 374–409 of 140K/98K was chemically synthesized and its secondary structure was then assessed using CD spectroscopy (Kelly and Price, 2000). CD spectra of the CTD peptide were recorded in aqueous phosphate buffer, or in the presence of 100% 2,2,2-trifluoroethanol (TFE)—a solvent mimicking the hydrophobicity of biological membranes, which stabilizes the folding of α-helical peptides (Rajan and Balaram, 1996), or in semihydrophobic solution (50% TFE). The spectra obtained (**Figure 5A**) all displayed distinct minima at 208 and 222 nm, implying that the CTD peptide adopts a predominantly α-helical fold (Greenfield, 2006). In aqueous buffer, the peptide readily adopted a helical conformation, but a gradual increase in this conformation was observed upon addition of increasing amounts of TFE as the helicity of the peptide, determined using different deconvolution algorithms (Whitmore and Wallace, 2008), ranged from 56 ± 3% in phosphate buffer, to 69 ± 7% in 50% TFE, and to 88 ± 9% in 100% TFE (**Figure 5B**).

Altogether, these results indicate that the 140K/98K CTD presumably folds within two α-helices, with predicted amphipathic properties, which can adopt an α-helical conformation in solution—a propensity that is even higher in a hydrophobic environment.

### The Amphipathic Nature of CTD α-Helices Is Required to Target 140K/98K to Chloroplasts *in Vivo*

We next investigated the contribution of αA and αB to the targeting of 140K/98K to chloroplasts *in vivo*.

We first addressed the importance of αA and αB *helical* structure by designing EGFP-140K mutants, which express altered proteins with amino acid residues Leu383 and Leu390 within αA and/or residues Leu401 and Leu404 within αB replaced by proline. The corresponding mutants were designated EGFP-140K-αA(LL/PP), EGFP-140K-αB(LL/PP) and EGFP-140K-αA-αB(LL/PP), respectively.

**FIGURE 6:**
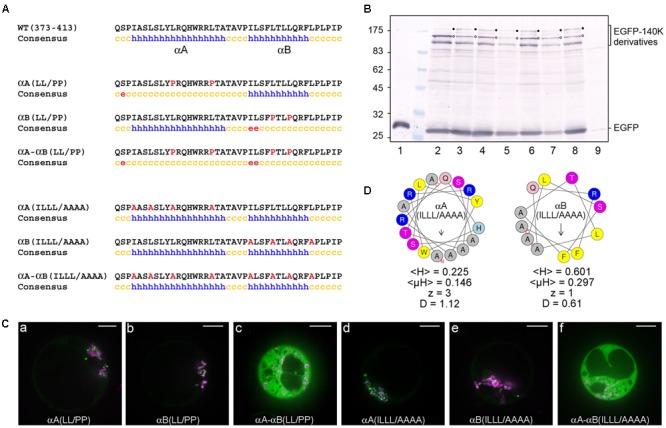
The structure and amphipathic property of CTD α-helices is required for targeting 140K/98K to the chloroplasts. **(A)** Sequence and secondary structure predictions of altered CTD domain. Introduced substitutions are highlighted in red in the residue sequence. Secondary structure predictions were made using the same predictors as in **Figure 4** but only the consensus prediction is shown (h: helix; c: coil; e: β-sheet). **(B)** Arabidopsis protoplasts were transfected with the expression plasmids pΩ-EGFP (lane 1), pΩ-EGFP-140K (lane 2), pΩ-EGFP-140K-αA(LL/PP) (lane 3), pΩ-EGFP-140K-αB(LL/PP) (lane 4), pΩ-EGFP-140K-αA-αB(LL/PP) (lane 5), pΩ-EGFP-140K-αA(ILLL/AAAA) (lane 6), pΩ-EGFP-140K-αB(ILLL/AAAA) (lane 7), pΩ-EGFP-140K-αA-αB(ILLL/AAAA) (lane 8), or with water (lane 9). The cells were harvested 48 hpt and equivalent amounts of total proteins (except lane 1 which corresponds to 1/20th of the other samples) were subjected to 10% SDS-PAGE and immunoblot analysis with anti-GFP antibodies. Molecular mass markers (Biolabs) are indicated on the left whereas positions of EGFP-140K derivatives and EGFP are indicated on the right. Filled dots indicate the position of full-length proteins, whereas open dots indicate the position of the mature product after processing at the (PRO↓HEL) cleavage site. **(C)** Arabidopsis protoplasts were transfected with the expression plasmids pΩ-EGFP-140K-αA(LL/PP) **(a)**, pΩ-EGFP-140K-αB(LL/PP) **(b)**, pΩ-EGFP-140K-αA-αB(LL/PP) **(c)**, pΩ-EGFP-140K-αA(ILLL/AAAA) **(d)**, pΩ-EGFP-140K-αB(ILLL/AAAA) **(e)** or pΩ-EGFP-140K-αA-αB(ILLL/AAAA) **(f)**, together with pΩ-RbCS-NiRFP. Single protoplasts were observed by spinning-disk confocal laser microscopy (SPCLM) 48 hpt and EGFP localization was observed (green). To visualize the localization of chloroplasts, NiRFP fluorescence and chlorophyll autofluorescence were acquired (magenta) and superimposed onto the EGFP fluorescence. Scale bars, 10 μm. **(D)** Helical wheel representation of αA(ILLL/AAAA) and αB(ILLL/AAAA) helices using the HeliQuest server. Color code and helix characteristics are the same as in **Figure 4**.

As expected from the potent helix-breaking property of a proline residue when present in the middle of a helical sequence, the secondary structure predictions of these altered proteins confirmed the disruption of the corresponding helices, alone or in combination (**Figure 6A**). The importance of each helix for the subcellular localization of 140K/98K was then investigated in Arabidopsis protoplasts transiently expressing the corresponding EGFP-140K mutants, expression of which was verified by western-blotting using anti-EGFP antibody (**Figure 6B**, lanes 3–5). Detection of EGFP-98K cleavage products (open dots) confirmed that the introduced substitutions did not impair processing of the EGFP-140K precursor proteins.

As shown in **Figures 6Ca,b**, disruption of each helix individually did not impede targeting of the fusion proteins to the chloroplast, whereas the simultaneous disruption of the two helices completely abolished localization to the chloroplasts, leading to a fully cytoplasmic protein (**Figure 6Cc**).

The importance of α-helices *amphipathy* in the subcellular localization of 140K/98K was subsequently tested by designing mutants which express altered proteins with changes within the hydrophobic face of each helix. Amino acid residues Ile376, Leu379, Leu383, and Leu390 within αA and/or residues Ile397, Leu401, Leu404, and Leu408 within αB were replaced with alanine residues to avoid interference with the structure or global charge of the CTD. The corresponding mutants were designated EGFP-140K-αA(ILLL/AAAA), EGFP-140K-αB(ILLL/AAAA) and EGFP-140K-αA-αB(ILLL/AAAA), respectively.

Although structure prediction still identified two putative α-helices within the resulting altered sequences (**Figure 6A**), those alanine substitutions caused a strong decrease in the mean hydrophobicity <H>, the hydrophobic moment <μH> and the discriminant factor D of each helix (**Figure 6D**), as compared to their wild-type counterpart (**Figure 4B**).

Upon transient expression of the corresponding mutants in Arabidopsis protoplasts (**Figure 6B**, lanes 6–9), observation of their subcellular localization revealed that alteration of the hydrophobic face of each single helix still allowed chloroplast targeting of the fusion proteins (**Figures 6Cd,e**), whereas combining substitutions in the hydrophobic face of both helices completely abolished localization to the chloroplasts, leading to a fully cytoplasmic localization (**Figure 6Df**). These results thus demonstrate the importance of the amphipathic properties of αA and αB α-helices for the targeting of 140K/98K to the chloroplasts. They also revealed the apparent redundancy of the two helices, as both required to be altered for the chloroplast subcellular targeting to be impaired.

**FIGURE 7:**
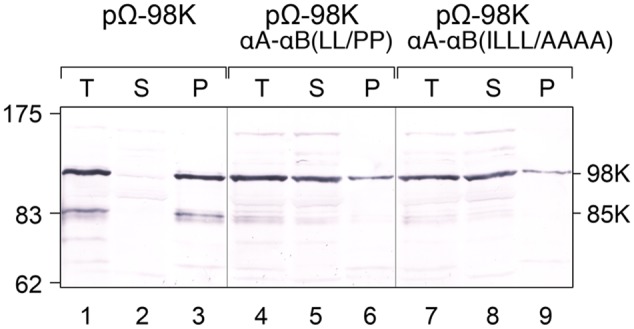
Impact of the structure and amphipathic property of CTD α-helices on membrane association of 98K *in vivo.* Arabidopsis protoplasts were transfected with the expression plasmids pΩ-98K, pΩ-98K-αA-αB(LL/PP) or pΩ-98K-αA-αB(ILLL/AAAA) as indicated. The cells were harvested 24 hpt and were lyzed to generate a total protein fraction (T). Soluble (S) and insoluble pellet (P) fractions were then separated by centrifugation, and samples of each fraction corresponding to equal amount of fresh tissue were subjected to 8% SDS-PAGE and immunoblot analysis using anti-98K antiserum. Molecular mass markers (Biolabs) are indicated on the left, whereas positions of the viral proteins 98K and 85K are indicated on the right.

The impact of amino acid residue substitutions in the CTD α-helices on the membrane association of 98K was then assessed biochemically by performing subcellular fractionation experiments via differential centrifugation. For that purpose, protoplasts expressing proteins 98K, 98K-αA-αB(LL/PP) or 98K-αA-αB(ILLL/AAAA) were lysed and, after removing cell debris by low-speed centrifugation, the total protein fraction was subjected to centrifugation at 25,000 × *g*, giving rise to a membrane pellet (P) and soluble (S) subcellular fractions. Samples of each fraction corresponding to an equal amount of fresh tissue were subsequently analyzed by western-blotting. As shown in **Figure 7** (lanes 1–3), wild-type 98K protein was found exclusively associated with the membrane pellet, consistent with its subcellular localization at the chloroplast envelope membrane. In contrast, although a minor fraction of the altered proteins was recovered in the pellet fraction, proteins 98K-αA-αB(LL/PP) (lanes 4-6) and 98K-αA-αB(ILLL/AAAA) (lanes 7–9) were predominantly present in the soluble fraction, indicating that the corresponding substitutions strongly affected their membrane association properties.

Altogether, these results indicate that both the helical structure and the amphipathic properties of αA and αB are required for targeting 140K/98K to the chloroplast envelope, and for membrane association of 98K *in vivo.*

### Effect of Mutations of the CTD on Viral Infectivity

To analyze whether the alterations in the CTD that affect chloroplast targeting are tolerated by the virus, the mutations αA-αB(LL/PP) and αA-αB(ILLL/AAAA)—shown in **Figures 6, 7** to impair chloroplast targeting of 140K/98K and membrane association of 98K—were introduced into a full-length cDNA clone of TYMV from which infectious viral transcripts can be obtained. To prevent the introduction of concomitant modifications in the overlapping 69K protein sequence (**Figure 1**), instead of using the WT E17 construct (Drugeon and Jupin, 2002), we rather chose to introduce the mutations into the construct E17-stop69K in which a stop codon truncates the 69K ORF at codon 30 (Prod’homme et al., 2003). Truncation of 69K was previously shown not to prevent viral replication or chloroplast targeting of the VRC (Prod’homme et al., 2003).

**FIGURE 8:**
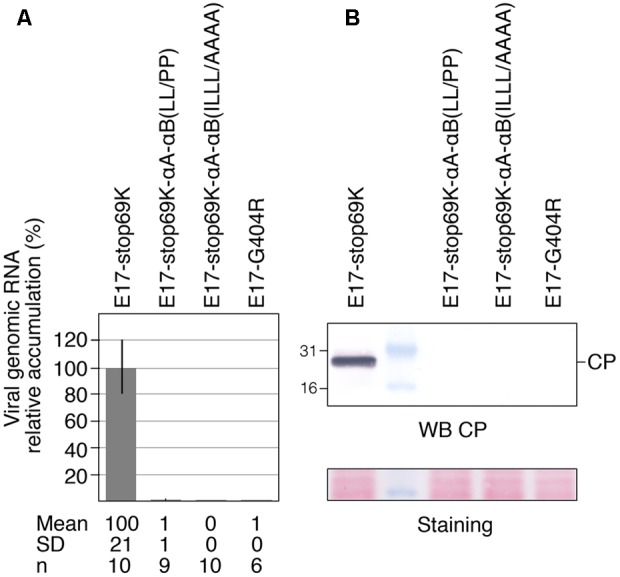
Impact of the structure and amphipathic property of CTD α-helices on viral infectivity. Arabidopsis protoplasts were transfected with *in vitro* transcripts as indicated, and cells were harvested 48 hpt. **(A)** The ability of the transcripts to replicate was assessed by extracting total RNAs and quantifying viral genomic RNA by RTqPCR. The relative accumulation of viral mutant RNAs as compared to the E17-stop69K control is represented as the mean ± SD. Mean and SD values, as well as the number of samples (n) analyzed in two independent experiments are indicated below panel **(A)**. **(B)** The ability of the transcripts to replicate was assessed by subjecting total protein samples to 15% SDS-PAGE and immunoblot analysis using anti-CP antiserum. Molecular mass markers (Biolabs) are indicated on the left whereas position of CP is indicated on the right. Ponceau staining of the membrane (staining) indicates protein loading.

Full-length RNAs were generated by *in vitro* transcription and equal amounts of *in vitro* transcripts were used to transfect Arabidopsis protoplasts. Viral infectivity was assessed by detecting viral genomic RNA progeny by RT-qPCR (**Figure 8A**) or capsid protein (CP) by Western blotting (**Figure 8B**), as the latter is dependent of viral replication for its expression from a subgenomic RNA. The parental transcript E17-stop69K was included as a positive control, while E17-G404R mutant, carrying a mutation within the ultra-conserved GDD motif in the polymerase catalytic domain (Jakubiec et al., 2006), served as a negative control. As shown in **Figures 8A,B**, no viral RNA replication could be detected for either CTD mutant, indicating that mutations that prevent 140K/98K chloroplast targeting by either disrupting αA-αB helix folding, or modifying their amphipathic properties, both have a dramatic impact on the function of 140K/98K in TYMV RNA replication.

**FIGURE 9:**
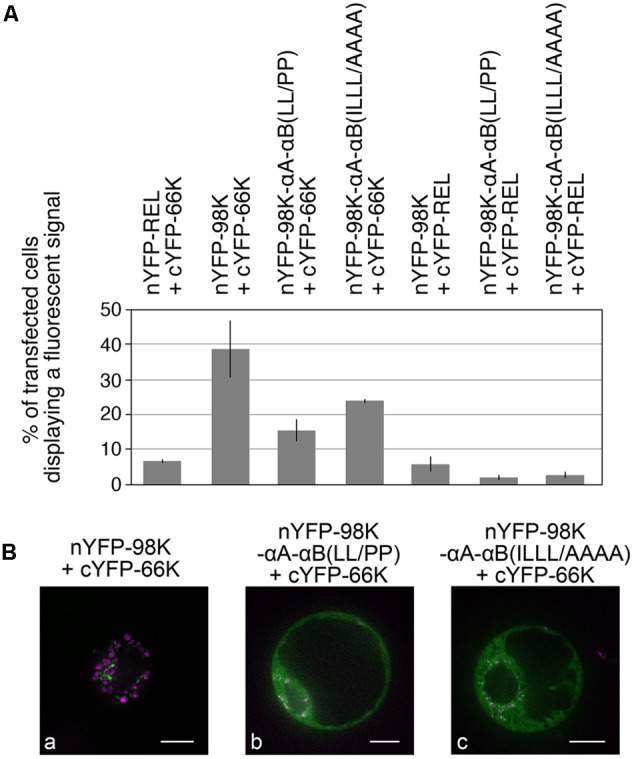
Impact of the structure and amphipathic property of CTD α-helices on the ability of 98K to interact with 66K as determined by bimolecular fluorescence complementation. **(A)** Arabidopsis protoplasts were cotransfected with the expression plasmids as indicated, together with pΩ-RbCS-NiRFP. Cells were collected 40 hpt and the percentage of cells displaying a fluorescent YFP signal was determined by flow-cytometry. Values were normalized to the percentage of transfected cells and are represented as the mean ± SD of three independent experiments. **(B)** Single protoplasts were observed by spinning-disk confocal laser microscopy (SPCLM) 48 hpt and YFP localization was observed (green). To visualize the localization of chloroplasts, NiRFP fluorescence and chlorophyll autofluorescence were acquired (magenta) and superimposed onto the YFP fluorescence. Scale bars, 10 μm.

To rule out a possible impact of such mutations on the ability of 98K to interact with the 66K polymerase, which is normally recruited to the replication complexes via protein–protein interaction with the PRO domain of 140K/98K (Jakubiec et al., 2004), we next performed bi-molecular fluorescence complementation (BiFC) assays to assess whether the interactions between TYMV replication proteins were still occuring *in vivo*. This approach relies on the generation of a fluorescent signal when two non-fluorescent fragments of YFP are brought close to each other, by virtue of interaction between two candidate proteins fused to these fragments (Walter et al., 2004). For that purpose, 98K and 66K were expressed in Arabidopsis protoplasts as fusion proteins with the N-terminal or C-terminal moieties of YFP, respectively (Citovsky et al., 2006). In order to determine the percentage of transfected cells displaying a fluorescent signal indicative of YFP reconstitution (i.e., interaction between the co-expressed proteins), transfected protoplasts were first analyzed by flow cytometry (Berendzen et al., 2012). As shown in **Figure 9A**, coexpression of nYFP-98K and cYFP-66K led to the detection of a fluorescent signal in ∼40% of transfected cells—a value markedly higher than the percentage of cells in which interaction of each partner protein with nYFP-REL or cYFP-REL used as negative controls was detected. A substantial proportion of fluorescent cells was also detected when cYFP-66K was coexpressed together with nYFP-98K-αA-αB(LL/PP) or nYFP-98K-αA-αB(ILLL/AAAA), indicating that the substitutions introduced in 98K CTD do not prevent their capacity to interact with 66K *in vivo* (**Figure 9A**). Further observation by confocal microscopy of cells displaying fluorescence revealed that interaction was detected at the periphery of chloroplasts in cells co-expressing nYFP-98K and cYFP-66K (**Figure 9Ba**), consistent with our previous observations (Prod’homme et al., 2003; Jakubiec et al., 2004), whereas coexpression of nYFP-98K-αA-αB(LL/PP) or nYFP-98K-αA-αB(ILLL/AAAA) with cYFP-66K led to the detection of a fluorescent signal throughout the cytosol (**Figures 9Bb,c**) consistent with the inability of both altered 98K proteins to be targeted to the chloroplast envelope (**Figure 6C**). These results therefore demonstrate that interaction between 98K and 66K is not dependent on chloroplast targeting of 98K, and rule out the possibility that the inability of viral mutants E17-stop69K-αA-αB(LL/PP) and E17-stop69K-αA-αB(ILLL/AAAA) to replicate may be caused by the impairment of 66K interaction with the corresponding altered 98K proteins.

## Discussion

The TYMV 140K protein was shown previously to be responsible for chloroplast targeting and recruitment of the polymerase to VRCs, as well as for some of the perturbations, such as chloroplast clumping, that are observed in infected cells. The molecular mechanisms by which the 140K protein targets chloroplast envelope membranes is largely unknown. In this study, we investigated the determinants of *in vivo* chloroplast targeting of this key viral replication protein.

Here, we present evidence that the CTD is located within a region shared by 140K and 98K (its mature cleavage product) so the question as to whether 140K or 98K, or possibly both, contribute to targeting of the VRCs still remains open. TYMV 98K behaves as a protein tightly associated to membranes, but not as a membrane-spanning protein. Furthermore, deletion studies indicate that a crucial domain required for chloroplast targeting of 140K/98K lies within the region of amino acids residues 373–413. Such domain appears sufficient to target GFP to the chloroplast envelope. This 41-residue-long domain was predicted to fold within two amphipathic α-helices—a folding that was confirmed *in vitro* using a synthetic peptide and CD analyses. The importance for the subcellular localization and function of 140K/98K of the integrity of these amphipathic helices was demonstrated by performing amino acid substitutions, which were shown to affect chloroplast targeting, membrane association and viral replication. From these data, we thus conclude that the two amphipathic helices αA and αB within 140K/98K constitute the determinants for chloroplast targeting of the TYMV VRCs.

### Amphipathic Helices as a Determinant for TYMV Replication Protein Targeting

The importance of amphipathic helices to act as membrane targeting and association determinants has been well established for a variety of proteins in both eukaryotes and prokaryotes (Segrest et al., 1990; Picot and Garavito, 1994; Johnson and Cornell, 1994; Thiyagarajan et al., 2004; Lu and Taghbalout, 2013).

In the case of replication proteins encoded by positive-strand RNA viruses, previous examples include picornavirus protein 2C (Echeverri and Dasgupta, 1995), alphavirus protein nsP1 (Ahola et al., 1999; Spuul et al., 2007), enterovirus protein 2B (de Jong et al., 2003), NS5A proteins of both hepaciviruses and pestiviruses (Brass et al., 2002, 2007), nepovirus NTB-VPg protein (Zhang et al., 2005), bromovirus protein 1a (Liu et al., 2009), hepacivirus NS4B protein (Gouttenoire et al., 2009a,b, 2014), or dianthovirus p27 protein (Kusumanegara et al., 2012).

In some cases, NMR spectroscopy-based structural analyses and molecular dynamics simulations have been reported (Lampio et al., 2000; Penin et al., 2004; Sapay et al., 2006; Liu et al., 2009; Gouttenoire et al., 2009a,b, 2014), revealing that the amphipathic helices identified in viral replication proteins can serve as membrane-anchoring domains by establishing in-plane interactions with the surface of the membrane, in a so-called monotopic interaction (Blobel, 1980). The hydrophobic residues can directly insert into membrane lipids, while the surrounding positively charged amino acids would further strengthen membrane binding by interacting with acidic phospholipid heads. The charged residues facing the cytosol were also proposed to serve as an assembly platform for intermolecular interactions with viral and/or host proteins essential for the functional architecture of the VRCs (Penin et al., 2004; Liu et al., 2009). This topology of viral replication proteins is consistent with our present understanding of the functioning of the VRCs, which require functional domains to be exposed unilaterally on one side of the membrane.

By analogy, given the importance of the hydrophobic face of the helices for subcellular targeting and membrane association, it is conceivable that both helices may serve as similar anchoring sequences through in-plane interactions with one of the two leaflets of the chloroplast outer envelope membrane. Such a model would be consistent with TYMV 98K being resistant to extraction with compounds that release weakly associated peripheral membrane proteins, while being substantially released from membranes by treatments with urea. In addition to the high content of Leu residues—the most common amino acid in the interface region of monotopic proteins—in the hydrophobic face of both helices, the presence of basic residues on the hydrophilic face of helices αA and αB also appears as another common feature of such oriented helices (Granseth et al., 2005). Moreover, Trp residues are known to be located preferentially at the lipid bilayer interface (Yau et al., 1998), and the location of Trp387 at the predicted interface between the hydrophilic and hydrophobic sides of helix αA (**Figures 4B,C**) is a very typical feature that strongly argues in favor of an in-plane interaction.

Future structural analyses of the CTD combined with mutagenesis studies will aim at probing the importance of these residues in the subcellular localization and membrane association properties of TYMV 98K to validate such hypotheses. We presently cannot rule out the possibility that the topology of the TYMV CTD might also be more complex, as reported in the case of HCV NS4B (Gouttenoire et al., 2014).

This isolated domain appears to target the correct membrane (**Figure 3C**), suggesting that the interactions of other regions within the protein are not critical for targeting. However, it is important to point out that our findings do not preclude the existence of other 98K sequences that may be important for anchoring to membranes, as those may be different from the determinants involved in subcellular targeting to chloroplasts *per se*. In that respect, **Figure 7** shows that a minor fraction of the altered 98K proteins was recovered in the membrane pellet. This may correspond to protein aggregates co-sedimenting with cellular membranes, or it may indicate that the altered proteins still had the ability to interact with membranes (other than the chloroplast envelope membranes based on the cell imaging observations), through another motif.

### A Targeting Signal Composed of Two α-Helices with a Semi-flexible Linker

Remarkably, the TYMV CTD appears constituted by two α-helices with apparent redundancy in their ability to target 140K/98K to the chloroplast envelope, as alteration of both helices was required to prevent localization to the chloroplast. Although each helix appeared to exhibit a sufficient number of membrane anchor residues (Trp, Phe, Leu and Ile), and proved sufficient to ensure the targeting to the chloroplast envelope membrane, such redundancy may be related to the fact that short amphipathic helices have a relatively weak affinity for membranes. Therefore, the involvement of several helices, oligomerization, or additional mechanisms such as lipid modification or positively charged segments are often involved in tightening membrane association of monotopic proteins (Johnson and Cornell, 1999).

In the case of viral replication proteins, having redundant/alternative strategies to maintain interactions with membranes may be even more crucial than for cellular proteins, given the high mutation rate of viral genomes, which may cause spontaneous mutations in replication proteins and impair the targeting, assembly or function of the VRCs upon which survival of the virus depends. For other positive-strand RNA viruses that have been studied, various strategies to tighten binding have been reported, for instance palmitoylation of Semliki Forest virus (SFV) nsP1 (Ahola et al., 2000), self-interaction between multiple peripherally located monomers of bromovirus 1a protein (den Boon et al., 2001), or the involvement of complex networks comprising several viral proteins, each containing both amphipathic and/or transmembrane helices, as in members of *Picornaviridae, Flaviviridae* or *Secoviridae* (Villanueva et al., 2005; den Boon and Ahlquist, 2010; Sanfaçon, 2012). The apparent redundancy between helices αA and αB of TYMV CTD may thus be envisaged as a “belt and braces” safety strategy to ensure proper targeting of the VRCs. However, at this stage, we cannot rule out the possibility that the two helices may play slightly different roles that have gone undetected with the cell biology approach used in this study.

Alternatively, because modeling experiments predicted that the two helices are separated by a short proline-containing linker that may contribute to the orientation/bending of the helices relative to each other, this situation may be advantageous over a longer helix, because of the conformational flexibility that it offers. This local bending allows restricted flexibility, which might be important to ensure the most favorable adaptation of the hydrophobic regions of the CTD to the specific physicochemical environment of the membrane interface. Indeed, internal helix bending and/or flexible interhelical loops appear as a common characteristic of in-plane membrane anchors of monotopic membrane proteins, as reported in several examples of cellular or viral proteins (Sapay et al., 2006).

Another—non-exclusive—possibility is that the linker might play a role in structural rearrangements of the CTD upon membrane binding. In this respect, it should be noted that the synthetic peptide corresponding to residues 374–409 of 140K/98K already displayed a significant helicity in aqueous solution as revealed by CD analyses, but that its propensity to adopt an α-helical conformation was even higher in a hydrophobic environment, suggesting an environment-dependent modulation of protein conformation. This partial folding in solution might be stabilized by intermolecular interactions between distinct peptides, or may reflect intramolecular interactions, with αA and αB folding onto each other in the absence of membranes, consistent with the proposed simulations (**Figure 4C**). Upon targeting of 140K/98K to the chloroplasts, these self-interactions may dissociate in favor of interactions with the membrane interface, promoting further helical folding of the CTD.

There are numerous data correlating protein conformational transitions and binding to lipids (Johnson and Cornell, 1999; Seelig, 2004). Given the importance of membranes and/or lipid composition for the assembly, activation and regulation of the VRCs (Wu et al., 1992; Ahola et al., 1999; Laliberté and Zheng, 2014; Nagy et al., 2016; Fernández de Castro et al., 2016; Altan-Bonnet, 2017), as well as the multiple functions played by TYMV 140K/98K (Rozanov et al., 1992, 1995; Kadaré et al., 1996; Prod’homme et al., 2003; Jakubiec et al., 2004, 2007; Chenon et al., 2012), it is tempting to speculate that CTD association with membranes may cause a conformational change in the structure of 140K/98K that may regulate some of its functions, as reported for SFV nsP1 (Ahola et al., 1999). However, direct evidence for a dynamic behavior of this region, and a possible impact on 140K/98K function, remains to be established.

### Location of the CTD in the Iceberg Region of the Methyltransferase Domain Supports Previous Theoretical Predictions

The CTD is located within a region of 98K/140K that had no attributed function in viral replication until recently, when it was proposed by Ahola and Karlin (Ahola and Karlin, 2015) to correspond to a C-terminal extension of the previously described methyltransferase-guanylyltransferase (MTase/GTase) domain (Rozanov et al., 1992). Despite the lack of sequence homology, extensive bioinformatics analyses and secondary structure predictions highlighted this region, which they refer to as the “Iceberg” region, as being present throughout the alphavirus-like supergroup of viruses. Their analysis revealed that the Iceberg region encompasses all the amphipathic helices known to promote membrane association of alphavirus and bromovirus VRCs (Lampio et al., 2000; Spuul et al., 2007; Liu et al., 2009). These helices appear phylogenetically distinct from each other, and also distinct from the TYMV CTD.

Interestingly, these authors also predicted that the Iceberg region may contain an overlooked, widely conserved, amphipathic helix with membrane-binding properties, both in the alto and tymo groups of the alphavirus-like supergroup. Although *Tymoviridae* were more divergent, it is striking to note that our data are in perfect agreement with their prediction, as the helix αA of the TYMV 140K/98K CTD indeed corresponds to the helix referred to as αI by Ahola and Karlin in the tymo group, and which they proposed to be involved in membrane binding (Ahola and Karlin, 2015). Our results thus constitute an experimental validation of their theoretical prediction, and further support the idea that the corresponding region may also be involved in membrane targeting of VRCs in other taxa.

### Preventing Chloroplast Targeting of 140K/98K Abolishes Viral Replication

We showed that introduction of mutations αA-αB(LL/PP) and αA-αB(ILLL/AAAA) into infectious transcripts led to complete loss of viral infectivity, demonstrating that the two amphipathic helices αA and αB play a key role in the early events of TYMV replication, and that 140K/98K proteins that are defective in chloroplast targeting are also severely affected in function.

We consider it unlikely that the defect in TYMV replication is caused by improper processing of the 206K precursor or its 140K intermediate cleavage product, as mature EGFP-98K proteins were detected upon expression of the altered EGFP-140K proteins in Arabidopsis protoplasts. We could also rule out the possibility that such alterations impaired the capability of 98K to interact with 66K polymerase, as demonstrated by BiFC experiments.

Because the Iceberg region in which the CTD is located was proposed to be essential for capping of the viral RNAs (Ahola and Karlin, 2015), it cannot be excluded that the introduced mutations may directly affect the MTase/GTase activities of TYMV 140K/98K. In that respect, it should be noted, however, that previous biochemical assays of MTase/GTase enzymatic activities were performed using *Bamboo mosaic virus* (BaMV) (Huang et al., 2004; Hu et al., 2011)—a potexvirus closely related to TYMV and which also belongs to the tymo group of the alphavirus-like supergroup. Such studies revealed that substitution of residues Trp377, Phe384 or Lys389 of BaMV replication protein, which are located in the predicted helices αI and αJ according to Ahola and Karlin’s nomenclature (Ahola and Karlin, 2015) (i.e., at positions corresponding to TYMV CTD helices αA and αB), had no effect on their enzymatic activities *in vitro* (Huang et al., 2004; Hu et al., 2011). Although direct evidence is still lacking in the case of TYMV, these data strongly support the idea that the replication failure of the CTD mutants is not linked to a defect in their capping activity, but rather to the inability of TYMV 140K/98K to be properly targeted to the chloroplast envelope membranes.

In that respect, it should be noted that although a minor fraction of 98K-αA-αB(LL/PP) and 98K-αA-αB(ILLL/AAAA) were recovered in the membrane pellet (**Figure 7**), whether they were still strongly membrane-associated and/or aggregated is unknown. However, the altered proteins were both non-functional, and, based on cell imaging observations, not detectably targeted to the chloroplasts, indicating that targeting to the proper organelles is essential for viral infectivity.

Such results are consistent with those previously obtained in other positive-strand RNA viruses (Moradpour et al., 2004; Spuul et al., 2007; Liu et al., 2009; Kusumanegara et al., 2012), and further confirm the importance of membrane targeting/association of viral replication proteins for the assembly of VRCs and viral RNA replication.

### The TYMV CTD Is an Unusual Targeting Signal for Chloroplast Outer Envelope Proteins

Most chloroplastic proteins are encoded by nuclear genes, translated on free polyribosomes in the cytosol, and targeted post-translationally to the organelle. Afterward, the recognition, translocation, and sorting of these proteins depends on the final destination of the protein within the various chloroplast sub-compartments. Whereas those imported into the chloroplasts rely mainly on a cleavable N-terminal transit peptide as a targeting signal and multisubunit protein complexes translocases (Jarvis and Robinson, 2004; Strittmatter et al., 2010; Kim and Hwang, 2013), those targeted to the chloroplast outer membrane do not possess a cleavable targeting signal and use alternative targeting pathways (Hofmann and Theg, 2005; Li and Chiu, 2010: Lee et al., 2014).

Recently, significant progress has been made in the identification of the signals and cytosolic events targeting proteins to the outer envelope membranes of chloroplasts. So far, three types of targeting signals have been identified : a N- or C-terminal transmembrane domain in the so-called signal- or tail-anchored proteins, respectively, or multiple transmembrane beta-strands in the so called β-barrel proteins (Lee et al., 2014). Therefore, the use of internal amphipathic helices such as those identified in the TYMV 140K/98K CTD appears to be a very unusual chloroplast envelope targeting signal that deserves particular mention, and whose detailed delivery mechanism remains to be elucidated.

In that respect, it should be noted that an increasing number of cellular proteins were recently reported to use a completely different and unexpected route, reaching the chloroplast via the secretory pathway, presumably via vesicle fusion with the organelle (Villarejo et al., 2005; Nanjo et al., 2006; Baslam et al., 2016). Viruses are well known for their capacity to exploit specific pathways in infected host cells in order to facilitate their replication, and the involvement of the coat protein complex II (COPII)-mediated vesicular transport pathway for the formation and translocation of VRCs-containing vesicles from the ER to chloroplasts has been well documented in the case of potyviruses (Wei and Wang, 2008; Wei et al., 2010, 2013). Whether TYMV replication proteins/VRCs also use this original pathway for their targeting to the chloroplasts is presently unknown.

In any case, one of the most challenging and intriguing questions concerning VRCs is how specific targeting mechanisms have been established, all the more so as different families of (+)RNA viruses utilize various subcellular membrane surfaces/organelles for replication. It is well known that organelle-specific lipids contribute to the unique identity of cellular compartments, enabling the sorting of proteins during membrane trafficking, and acting as receptors for the recruitment of specific enzymes and signaling molecules (van Meer et al., 2008; Klose et al., 2013). It is thus very likely that protein/lipid interactions contribute predominantly to the specificity of replication proteins/VRCs targeting (Lampio et al., 2000; Xu and Nagy, 2017; Altan-Bonnet, 2017), although stabilization by specific host factors cannot be excluded at this stage.

In this respect, it should be noted that chloroplast envelope membranes contain unique lipids, such as sulpholipids and, most importantly, the galactolipids mono- and digalactosyldiacylglycerol (MGDG and DGDG, respectively) (Joyard et al., 1991), which have been shown to play a critical role in protein targeting and binding to the chloroplast outer envelope (Bruce, 1998; Kim et al., 2014; Sarkis et al., 2014). In turn, binding of small peptides, particularly amphipathic helices, may promote changes in lipid organization and modulate membrane bilayer properties. Some amphipathic helices do not act as simple membrane anchors, but can also sense membrane curvature or deform lipid membranes (Drin and Antonny, 2010), thereby possibly contributing to the formation and size of the membrane vesicles hosting VRCs, as demonstrated in the case of BMV or HCV (Liu et al., 2009; Gouttenoire et al., 2014). It is possible that common mechanisms underlie these events in the case of TYMV.

## Conclusion

Our experiments provide a first characterization of the TYMV 140K/98K protein domain involved in targeting of replication complexes to the chloroplast envelope. Much work remains before we have a clear understanding of the steps involved in targeting of the CTD from the cytoplasm to the chloroplast outer surface, and how targeting of the CTD may be coupled to downstream events mediating its anchoring into the membrane bilayer and possible changes in protein structure or membrane bilayer properties linked to the formation of functional VRCs. However, delimitation of the CTD to a short peptide sequence will be an excellent starting point for a detailed investigation of its molecular interaction with membranes, and we hope that coupling biophysical and structural analyses of artificially reconstituted systems to biochemistry, *in vivo* cell imaging, and genetics may help clarify the mechanisms involved in these complex processes.

## Author Contributions

IJ conceived and coordinated the study. LM, LJ, and IJ designed, performed and analyzed the experiments. SB performed the modeling predictions. LM, LJ, SB, CA-L, and IJ contributed to the preparation of the Figures, drafting and revision of the manuscript. IJ wrote the manuscript. All authors reviewed the results and approved the final version of the manuscript.

## Conflict of Interest Statement

The authors declare that the research was conducted in the absence of any commercial or financial relationships that could be construed as a potential conflict of interest.

## Footnotes

1 http://dichroweb.cryst.bbk.ac.uk/html/process.shtml

2 http://www.cbs.dtu.dk/services/TargetP/

3 https://npsa-prabi.ibcp.fr/cgi-bin/npsa_automat.pl?page=/NPSA/npsa_seccons.html

4 http://bioinf.cs.ucl.ac.uk/psipred/

5 http://bioserv.rpbs.univ-paris-diderot.fr/services/PEP-FOLD3/

6 http://www.cbs.dtu.dk/services/TMHMM/

7 http://www.ch.embnet.org/software/TMPRED_form.html

8 http://mendel.imp.ac.at/sat/DAS/DAS.html

9 http://www.enzim.hu/hmmtop/

10 http://harrier.nagahama-i-bio.ac.jp/sosui/sosui_submit.html

11 http://heliquest.ipmc.cnrs.fr/

12 http://gpcr2.biocomp.unibo.it/gpipe/index.htm

13 http://lipid.biocuckoo.org/webserver.php
