# Supplementary material for: Identification and Molecular Characterization of the Chloroplast Targeting Domain of Turnip yellow mosaic virus Replication Proteins
Source: Front Plant Sci. 2017 Dec 19;8:2138. doi: 10.3389/fpls.2017.02138 (PMC5742235; doi:10.3389/fpls.2017.02138)
Supplement: Supplementary file 2 [file Image_1.pdf]

A

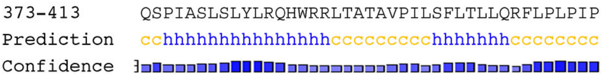

B

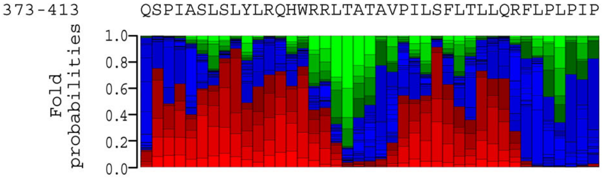

C

Predictor	Number of predicted TM	Residues
TMHMM	0	
TM-Pred	1	390-413 or 389-405
DAS-TM	1	397-406
HMMTOP	1	390-408
SOSUI	0	

D

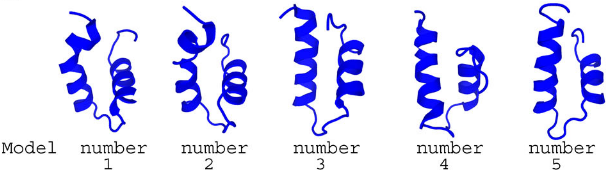

Supplementary Figure 1

(A) Secondary structure prediction of the CTD domain within the 140K/98K protein using the PSI-PRED predictor (Mc Guffin et al., 2000).
The sequence in one-letter code is shown at the top. c= coil, h= helix.The confidence value of the prediction at each position is represented as a blue bar graph below the prediction.
(B) Secondary structure local prediction of the CTD domain within the 140K/98K protein using the PEP-FOLD predictor (Camproux et al., 2004) based on structural alphabet (SA) prediction profile.
The probabilities at each position of the sequence, of the 27 SA letters, are sorted from helical (red), coil (blue) to extended (green) and are indicated on the left.
(C) Predictions of membrane-spanning regions using the predictors TMHMM (Krogh et al, 2001), TM-Pred (Hofmann & Stoffel, 1993), DAS-TM (Cserzo et al, 2004), HMMTOP (Tusnády & Simon, 2001), and SOSUI (Hirokawa et al, 1998).
The number of transmembrane segment predicted and their corresponding position within the CTD domain of 140K/98K protein is indicated.
(D) Representations of the 5 top models of de novo 3D peptide structure modeling of the CTD peptide using PEP-FOLD 3 (Lamiable et al, 2016). The top model n°1 corresponds to the one presented in Fig. 4C.
